# Activated CD4^+^ T cells-derived exosomal miR-142-3p boosts post-ischemic ventricular remodeling by activating myofibroblast

**DOI:** 10.18632/aging.103084

**Published:** 2020-04-23

**Authors:** Lidong Cai, Gong Chao, Weifeng Li, Jumo Zhu, Fangfang Li, Baozhen Qi, Yong Wei, Songwen Chen, Genqing Zhou, Xiaofeng Lu, Juan Xu, Xiaoyu Wu, Guangjian Fan, Jun Li, Shaowen Liu

**Affiliations:** 1 Department of Cardiology, Shanghai General Hospital, Shanghai Jiao Tong University School of Medicine, Hongkou District, Shanghai 201620, China; 2 Institute of Translational Medicine, Shanghai General Hospital, Shanghai Jiao Tong University School of Medicine, Shanghai 200080, China; 3 Department of Cardiology, Shanghai Institute of Cardiovascular Disease, Zhongshan Hospital, Fudan University, Shanghai 200080, China

**Keywords:** cardiac fibrosis, myocardial infarction, CD4 T cells ^+^, exosome, miR-142-3p

## Abstract

Cardiac fibrosis is a primary phenotype of cardiac remodeling that contributes to cardiac dysfunction and heart failure. The expansion and activation of CD4^+^ T cells in the heart has been identified to facilitate pathological cardiac remodeling and dysfunction; however, the underlying mechanisms remained not well clarified. Herein, we found that exosomes derived from activated CD4^+^ T cells (CD4-activated Exos) evoked pro-fibrotic effects of cardiac fibroblasts, and their delivery into the heart aggravated cardiac fibrosis and dysfunction post-infarction. Mechanistically, miR-142-3p that was enriched in CD4-activated Exos recapitulated the pro-fibrotic effects of CD4-activated Exos in cardiac fibroblasts, and vice versa. Furthermore, miR-142-3p directly targeted and inhibited the expression of Adenomatous Polyposis Coli (APC), a negative WNT signaling pathway regulator, contributing to the activation of WNT signaling pathway and cardiac fibroblast activation. Thus, CD4-activated Exos promote post-ischemic cardiac fibrosis through exosomal miR-142-3p-WNT signaling cascade-mediated activation of myofibroblasts. Targeting miR-142-3p in CD4-activated Exos may hold promise for treating cardiac remodeling post-MI.

## INTRODUCTION

Myocardial infarction (MI) represents a serious cardiovascular event, and accounts for a leading cause of morbidity and mortality worldwide [1]. Great advancements in pharmacological and interventional therapies have been made to rescue the dying myocardium, but cardiac remodeling post-MI, especially ventricular fibrosis, remains still a major challenge in clinical practice [2, 3]. There is an unmet need to identify novel mechanisms and molecular entities underlying the fibrotic remodeling post-MI.

Emerging evidences point to a potential link between immune and pathological cardiac remodeling [4, 5]. Sterile inflammation triggered by myocardial necrosis initiates cardiac repair post-MI [4, 6], whereas the sustained inflammation that is particularly mediated by cardiac infiltration of CD4^+^ T-cells has been implicated in the progression of cardiac fibrosis and dysfunction [7]. Immune therapeutics targeting CD4^+^ T cells protect the ischemic heart against fibrotic pathology and dysfunction [8]. Indeed, the deleterious effects of cardiac infiltration of CD4^+^ T cells have also been witnessed in pressure overload-induced cardiac hypertrophy and fibrosis, which could be depressed by genetic inactivation of CD4^+^ T cells [9]. These findings highlight the contributions of cardiac activated CD4^+^ T cells to maladaptive cardiac remodeling, but the underlying mediators await further elucidation.

Exosomes can be released by almost all types of cells, and modulate intercellular communication through transferring proteins, mRNA, and miRNA between cells [10]. They have the potential for cell-specific targeting [11], and usually hijack the trajectories of various pathological progressions [12–14], including cancer, viral infection, and amyloidopathies. In the cardiovascular field, exosomes are important regulators of various cardiovascular diseases [15], besides the roles as biomarkers [16]. For example, exosomes secreted by mesenchymal stem cell protect the heart against ischemia-reperfusion injury [17]. Cardiac fibroblast-derived exosomes facilitate pathological cardiac hypertrophy via activating renin angiotensin system in cardiomyocytes [18], and exosomes-derived from cardiomyocytes contribute to cardiac fibrogenesis via myocyte-fibroblast cross-talk [19]. Obviously, the cell source of exosomes greatly determines the action modality and functional outcome of exosomes.

Given that CD4^+^ T cell activation promotes pathological cardiac remodeling and that immune cells can release exosomes [20–22], we hypothesized that activated CD4^+^ cells-derived exosomes deteriorated cardiac fibrosis post-MI. We identified that CD4^+^ T cell-derived exosomes could be uptaken by cardiac fibroblasts and whereby contributed to cardiac fibroblast transformation. Furthermore, miR-142-3p-enriched in the activated CD4^+^ cell exosomes targeted Adenomatous Polyposis Coli (APC) to activate the WNT signal pathway, perpetuating myofibroblast activation and fibrogenesis.

## RESULTS

### Exosomes derived from activated CD4+ T cells promote cardiac fibroblasts activation

**Figure 1:**
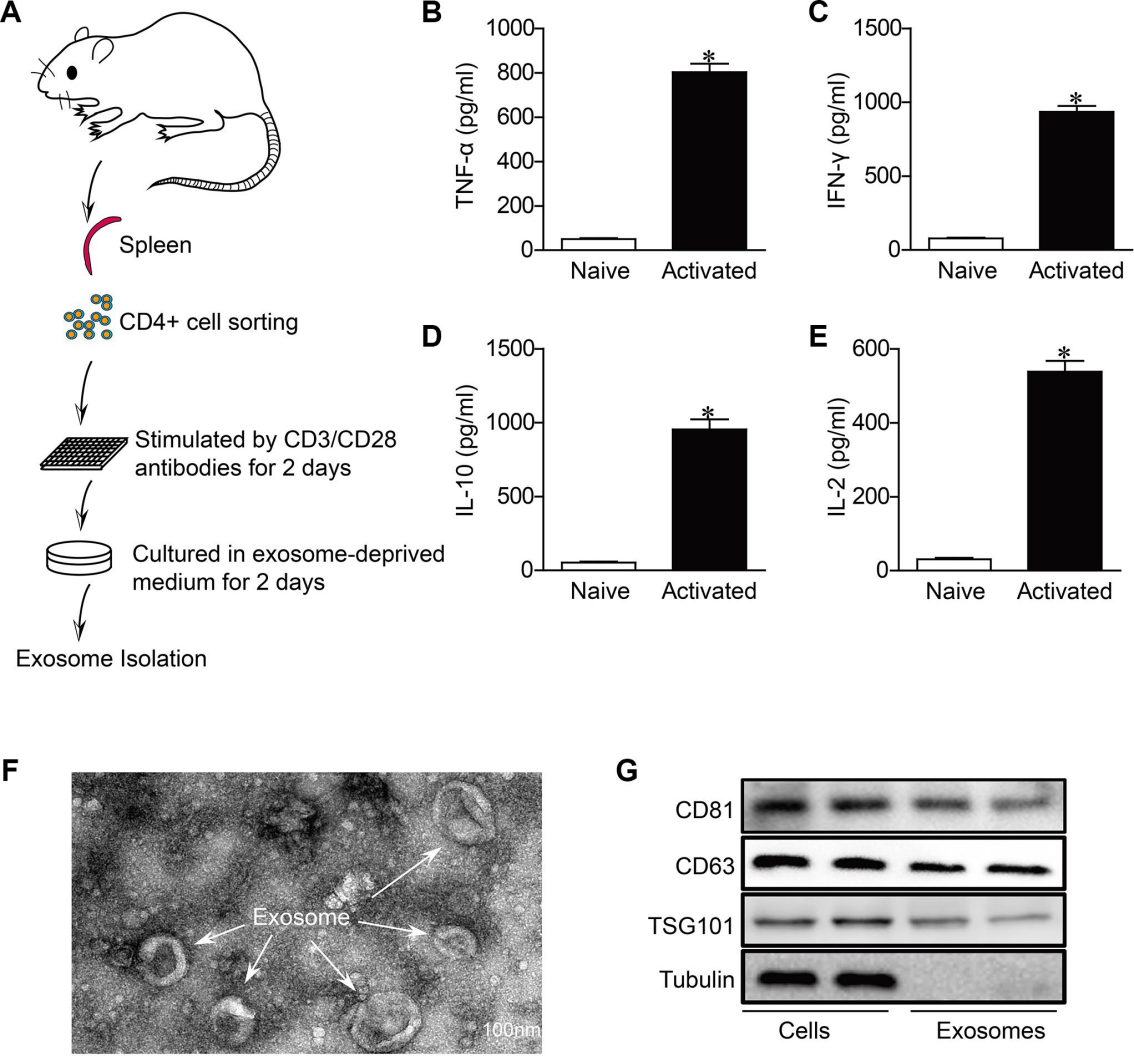
**Characterization of activated CD4+ T cells-derived exosomes.** (**A**) Schematic diagram for the isolation procedure of activated CD4^+^ T cells-derived exosomes. (**B**–**E**) ELISA analysis of IFN-γ,TNF-α, IL-2 and IL-10 in CD4^+^ T cells in response to anti-CD3 and anti-CD28 antibodies treatment for 48h (n = 5). *P < .05. (**F**) Transmission electron microscopic images of adult mouse CD4^+^ T cells-derived exosomes. Scale bar = 100 nm. The image shown is representative of three independent experiments. (**G**) Western blotting examination of exosome biomarkers in CD4^+^ T cells exosomes. The blots shown are representative of three independent experiments.

Consistent with previous studies [23], we found that CD4^+^ T cells remarkably infiltrated in the heart of myocardial infarction (Supplementary Figure 2). As CD4^+^ T cells infiltration and activation in the heart have been proven to worsen the development of non-infectious myocardial diseases [7, 8]. we wondered that whether exosomes derived from activated CD4+ cells (Exos-activated) contribute to cardiac remodeling post-MI. For this end, we isolated CD4+ T cells from mouse spleen, and stimulated with CD3/CD28 antibodies for 2 days to induce the activation of CD4+ cells (Figure 1A). As CD4^+^ T cells were predominantly differentiated into T helper cell type 1 in the presence of CD3/CD28 antibodies [24], we thus detected pro-inflammatory cytokines in culture media. The expression of TNF-α, IFN-γ, IL-10 and IL-2 were significantly upregulated, indicating the activation of CD4^+^ cells (Figure 1B–1E).

We then isolated exosomes from the conditions medium of CD+4 cells. TEM revealed that the predominant vesicles were of typical exosomal size (30-150 nm in diameter) with the characteristic round or “cup-shaped” delineated by a lipid bilayer (Figure 1F), which is consistent with previous reports [25]. Western blot analysis demonstrated the expression of CD81, CD63 and tumor susceptibility gene 101 (TSG101), which are all exosome markers and associated with exosome formation [26], in both exosomes and cells (Figure 1G).

**Figure 2:**
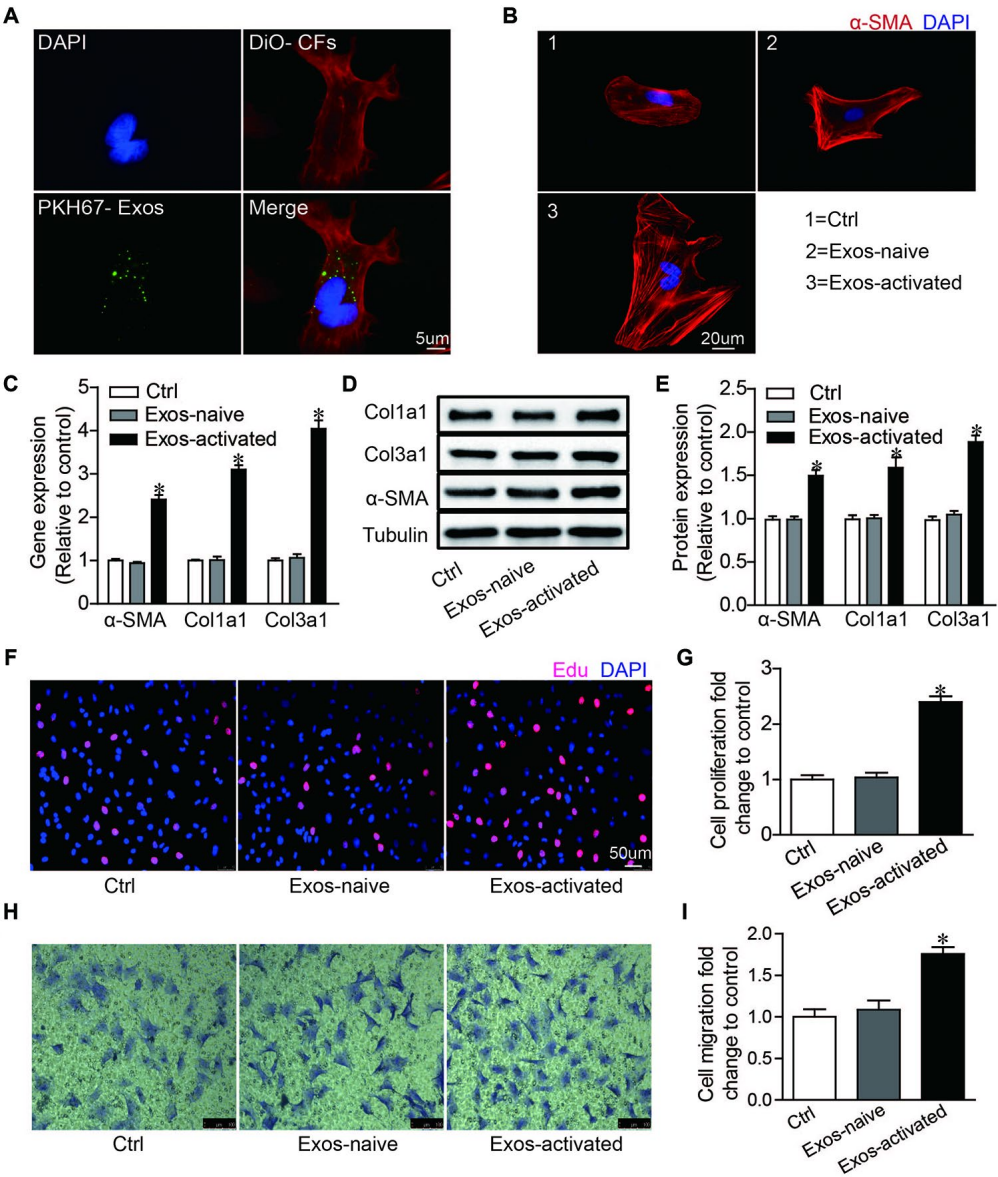
**Activated CD4^+^ T cells-derived exosomes promote cardiac fibroblasts activation in vitro.** (**A**) Immunofluorescence imaging analysis PKH67-labeled exosomes were taken up by cardiac fibroblasts. Green: exosomes; Red: cardiac fibroblasts; Blue: DAPI. The images shown are representative of three independent experiments. Scale bar = 5 μm. (**B**) Immunofluorescent analysis of myofibroblast activation. Red signals indicated α-SMA protein expression, and blue signals for nuclei. The images shown are representative of three independent experiments. Scale bar = 20 μm. Ctrl: control. Exos-naive: exosomes derived from naive CD4^+^ cells. Exos-activated: exosomes derived from activated CD4^+^ cells. (**C**) qPCR analysis of α-SMA, Col1α1 and Col3α1 levels in cardiac fibroblasts incubated with activated CD4^+^ T cells-derived exosomes for 48h. n = 3 per group. The blots shown are representative of three independent experiments. *P < .05 vs Exos-naive. (**D**) Expression levels of α-SMA, Col1a1 and Col3a1 were detected by western blot analysis. (**E**) Quantitative analysis of proteins expression using Image J software. *P < .05 vs Exos-naive. (**F**) EdU incorporation detection of cardiac fibroblast proliferation. The images shown are representative of three independent experiments. Scale bar = 50 μm. (**G**) Quantification analysis of cell proliferation using EdU assay data. *P < .05 vs Exos-naive. (**H**) Transwell assay of cardiac fibroblast migration. The images shown are representative of three independent experiments. Scale bar = 100 μm. (**I**) Quantification analysis of cell migration using transwell assay data. *P < .05 vs Exos-naive.

To explore the effects of Exos-activated on cardiac fibroblasts (CFs), we labeled exosomes with PKH-67 green dye and incubated these PKH67-labeled exosomes (PKH-67 are lipophilic cell tracking dyes with long aliphatic tails into lipid regions of exactly lipophilic on exosomes) with CFs for 2 h. The fluorescent imaging showed that exosomes could be endocytosed by CFs (Figure 2A). Moreover, we found that the Exo-activated could remarkably induce CFs differentiation, proliferation and migration (Figure 2B–2I). These findings indicated that exosomes derived from activated CD4+ T cells evoked fibrogenic behaviors of CFs.

### Exosomes derived from activated CD4^+^ T cells aggravate cardiac dysfunction post-MI

**Figure 3:**
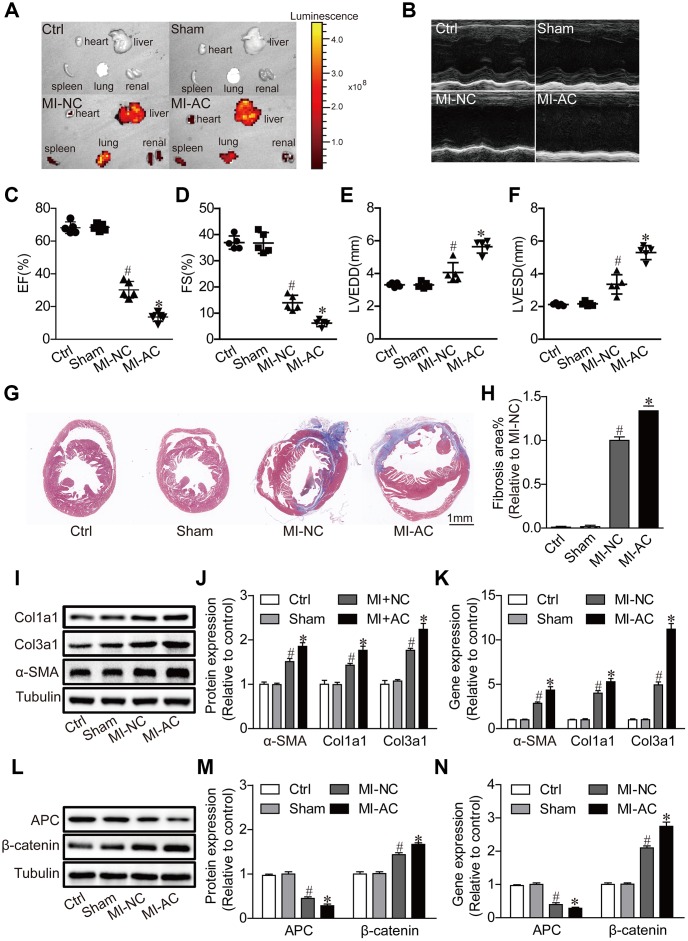
**Activated CD4^+^ T cells-derived exosomes deteriorate cardiac function post-MI in mouse.** (**A**) *Ex vivo* fluorescence imaging of major organs from mice. MI-NC: mice underwent myocardial infarction and injected with DiO-labeled naive CD4^+^- exosomes by by tail vein. MI-AC: mice underwent myocardial infarction and injected with DiO-labeled activated CD4^+^- exosomes by by tail vein. (**B**) Representative echocardiography at the fourth week post-MI. n = 5 per group. (**C**–**F**) Statistic summary from (**B**). EF: ejection fraction; FS: fractional shortening; LVESD: left ventricular end-systolic dimension; LVEDD: left ventricular end-diastolic dimension (n = 5). #P < .001 vs Sham. *P < .05 vs MI-NC. (**G**, **H**) Masson's trichrome staining of the cross section of the heart and quantification of the total fibrotic area using Image J software. The images shown are representative of three independent experiments. n = 5 per group. Scale bar = 1mm. #P < .001 vs Sham; *P < .05 vs MI-NC. (**I**) Expression levels of α-SMA, Col1a1 and Col3a1 were detected by western blot analysis. The blots shown are representative of three independent experiments. (**J**) Quantitative analysis of proteins expression of -SMA, Col1a1 and Col3a1 using Image J software. #P < .001 vs Sham; *P < .05 vs MI-NC. (**K**) qPCR analysis of α-SMA, Col1a1 and Col3a1 levels in the myocardium. n=3 per group. #P < .001 vs. Sham; *P < .05 vs. MI-NC. (**L**) Western blotting examination of APC and β-catenin protein expression. The blots shown are representative of three independent experiments. (**M**) Quantitative analysis of proteins expression of APC and β-catenin using Image J software. #P < .001 vs Sham; *P < .05 vs MI-NC. (**N**) qPCR analysis of APC and β-catenin levels in the myocardium. n=3 per group. #P < .001 vs. Sham; *P < .05 vs. MI-NC.

To further validate the pro-fibrotic effects of Exos-activated in vivo, Exos-activated were intravenously injected in the mice subject to MI. The significant fluorescence in the heart at the fourth week post-MI (Figure 3A) indicated the delivery efficacy of exosomes. Echocardiographic analysis revealed that the Exos-activated induced deterioration of cardiac dysfunction post-MI, as indicated by the smaller fractional shortening (FS%) and ejection fraction (EF%), and the larger left ventricular end diastolic diameter (LVEDD) and left ventricular end systolic diameter (LVESD) (Figure 3B–3F). Masson's trichrome staining of heart sections showed the larger dimension of left ventricles and more serious intestinal fibrosis in MI-AC group than in MI-NC group (Figure 3G, 3H). Accordingly, we found that the protein and mRNA expression of α-SMA, Col1α1 and Col3α1 were significantly enhanced in MI-AC group (Figure 3I–3K). Besides, the expression of APC and β-catenin in the four groups was checked, with the results that CD4-activated Exos upregulated the expression of APC and decreased β-catenin (Figure 3L–3N). These results indicated that canonical WNT signaling pathway might involve Exos-activated mediating cardiac fibrosis and dysfunction post-MI.

### MiR-142-3p critically mediates pro-fibrotic effects by CD4^+^ T cell-derived exosomes

**Figure 4:**
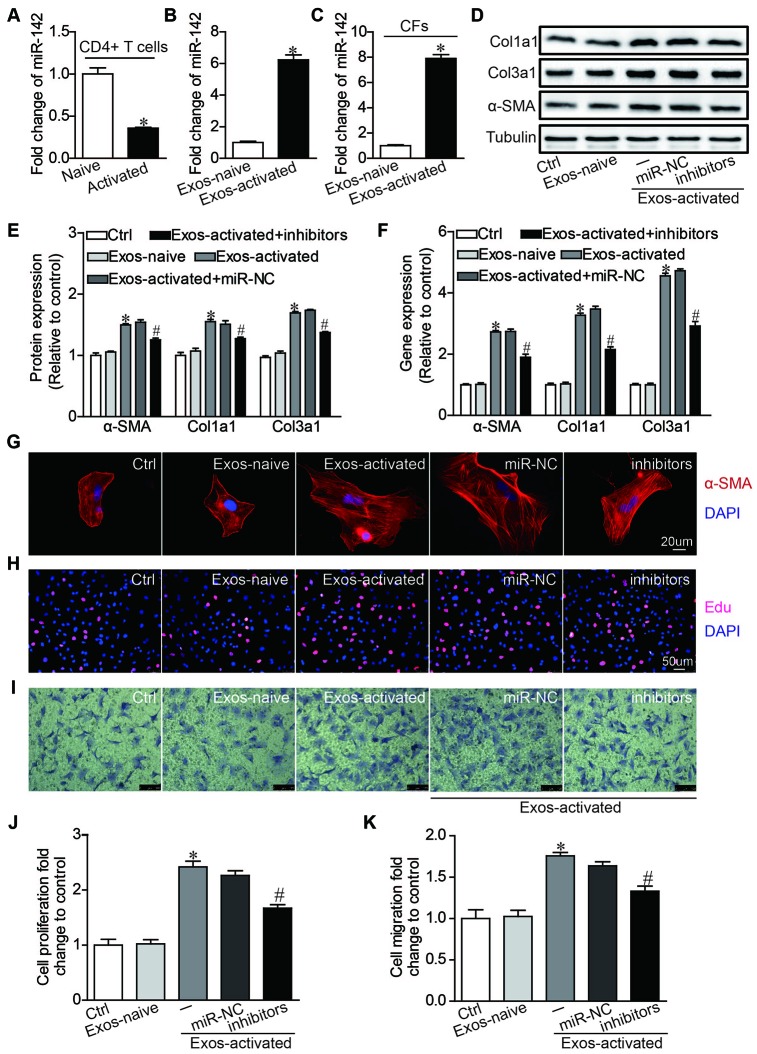
**MiR-142 partially mediated the pro-fibrotic effects of activated CD4^+^ T cells-derived exosomes on cardiac fibroblasts.** (**A**) MiR-142-3p expression was detected in naive CD4+ T cells and activated CD4+ T cells by qRT-PCR. n=3 per group. *P < .05. (**B**) MiR-142-3p expression was detected in exosome derived from naive and activated CD4+ T cells by qRT-PCR. n=3 per group. *P < .05. (**C**) MiR-142-3p expression was detected in CFs before and after incubated with exosomes derived from activated CD4+ T cells for 24h by qRT-PCR. n=3 per group. *P < .05. (**D**–**F**) Western blotting and qPCR analysis of α-SMA, Col1a1 and Col3a1 levels in cardiac fibroblasts. The blots shown are representative of three independent experiments. *P < .05 vs. Exos-naive. #p < .05 vs Exos-activated + miR-NC. (**G**) Immunofluorescent analysis of myofibroblast activation. The images shown are representative of three independent experiments. Red signals indicated α-SMA protein expression, and blue signals for nuclei. Scale bar = 20 μm. (**H**) Cardiac fibroblasts proliferation was detected using the EdU incorporation assay. The images shown are representative of three independent experiments. Scale bar = 50 μm. (**I**) Cardiac fibroblasts migration was detected using the transwell assay. The images shown are representative of three independent experiments. Scale bar = 100 μm. (**J**) Quantification analysis of cardiac fibroblasts proliferation using EdU assay data. *P < .05 vs. Exos-naive; #p < .05 vs. Exos-activated + miR-NC. (**K**) Quantification analysis of cardiac fibroblasts migration using Transwell assay data. *P < .05 vs. Exos-naive. #p < .05 vs. Exos-activated + miR-NC.

To further dissect the molecular mediator in Exos-activated for cardiac fibrosis post-MI, we focused on microRNAs that emerge as a novel functional carrier of exosomes [27]. MiR-142-3p is highly expressed in CD4+ T cells [28], and a recent study found that miR-142-3p is enriched in the exosomes derived from activated CD4^+^ T cells [29]. Thus we went on to ask whether miR-142-3p mediated the effects of Exo-activated on fibrogenic behaviors of CFs. qRT-PCR showed that miR-142-3p was downregulated in activated CD4^+^ T cells stimulated by anti-CD3/CD28 antibodies (Figure 4A), but it was upregulated in Exo-activated compared with exosomes derived from naive CD4+ T cells (Figure 4B). Strikingly, the level of miR-142-3p within CFs was remarkably upregulated after incubated with Exo-activated for 24h (Figure 4C). Next, to test whether the pro-fibrotic effects could be induced by exosomal miR-142-3p, CFs were transfected with miR-142-3p mimics. We found that miR-142-3p recapitulated the effects induced by Exo-activated, showing the differentiation and enhanced proliferation and migration of CFs (Supplementary Figure 3A–3E).

Then we validated the biological effects of miR-142-3p in CFs with its inhibitors. As opposite to miR-142-3p mimics, application of miR-142-3p inhibitors partially counteracted the pro-fibrotic effects of Exo-activated (Figure 4D–4K). Collectively, miR-142-3p is an important mediator for Exo-activated to evoked fibrogenic behaviors of CFs.

### MiR-142 directly targets the canonical WNT signaling pathway

**Figure 5:**
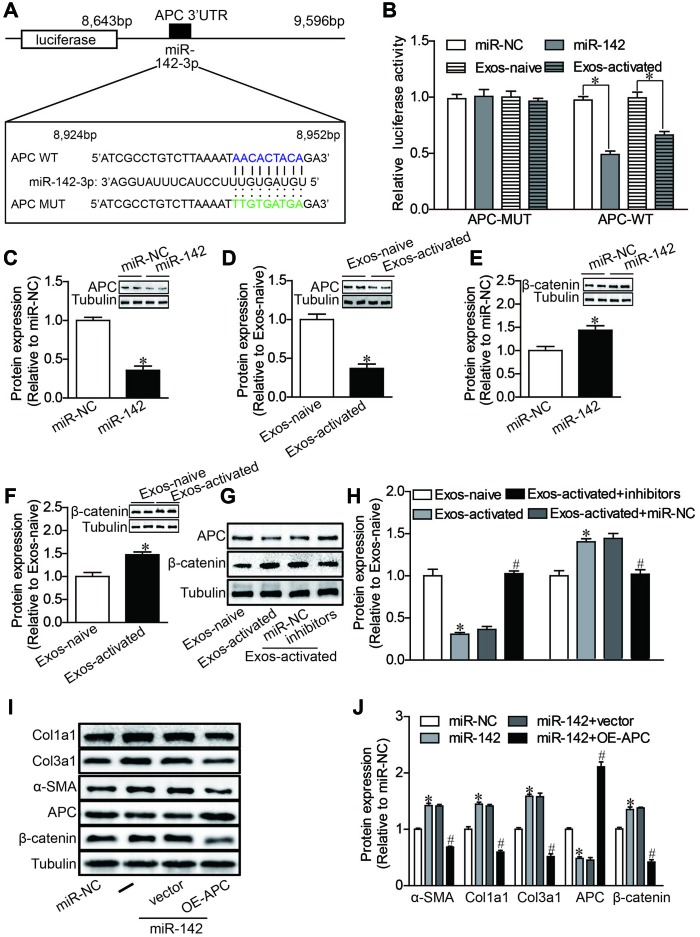
**MiR-142 targets APC, resulting in the activation of WNT pathway.** (**A**) Diagram of miR-142-3p binding site in APC 3′UTR. (**B**) Luciferase reporter assay of the interaction between miR-142-3p and APC. miR-142-3p overexpression and CD4-activated Exos treatment decreased the reporter activity in 293 T cells expressing the APC-Wt rather than APC-Mut vectors. n = 3 per group. *P < .05. (**C**–**H**) Western blot analysis of APC and β-catenin proteins. miR-142-3p overexpression and CD4-activated Exos treatment decreased the expression of APC and upregulated the expression of β-catenin in cardiac fibroblasts. miR-142-3p inhibitors reversed the effects of CD4-activated Exos on the expression of APC and β-catenin in cardiac fibroblasts. The blots shown are representative of three independent experiments. *P < .05. (**I**, **J**) Western blot analysis of α-SMA, Col1a1, Col3a1, APC and β-catenin proteins. APC overexpression reversed the upregulation of β-catenin expression, and the profibrotic effects of miR-142-3p in CFs. *P < .05 vs. miR-NC. #p < .05 vs miR-142-3P+ vector. vector: pcDNA3.1-NC, OE-APC:pcDNA3.1-APC.

Finally, we proceeded to elucidate the mechanisms underlying the pro-fibrotic effects of miR-142-3p. Using bioinformatics-based prediction, we found that miR-142-3p potentially targets Adenomatous Polyposis Coli (APC) at its 3′-UTR (Figure 5A). Dual luciferase reporter assay results indicated that miR-142-3p and Exos-activated significantly suppressed the relative luciferase activity of pmirGLO-APC-3′UTR compared with the control (Figure 5B). Western blotting also revealed that miR-142-3p overexpression and Exos-activated treatment decreased the expression of APC in CFs (Figure 5C, 5D). Given that APC protein acts as an important regulator for canonical WNT signaling pathway [30, 31], the expression of β-catenin, a core mediator of this signal cascade, was further tested in cardiac fibroblasts. We found that either miR-142-3p or Exos-activated remarkably enhanced the expression of β-catenin (Figure 5E, 5F), and conversely miR-142 inhibitors reversed the effects of Exos-activated on the expression of APC and β-catenin in cardiac fibroblasts (Figure 5G, 5H). To further validate whether miR-142 exerts a profibrotic effect by targeting APC, we co-transfected CFs with APC overexpression plasmid (1 ng) and miR-142 mimics (5nM). Overexpression of APC abolished the upregulation of β-catenin expression, and reversed the upregulation of alpha-SMA, Col1a1 and Col3al expression induced by miR-142-3p in CFs (Figure 5I, 5J). All these results indicated that activated CD4^+^ T cells promoted cardiac myofibroblast activation through exosome-enriched miR-142-3p-WNT signaling cascade.

## DISCUSSION

The mechanisms by which activated CD4^+^ T cells contribute to heart failure progression remain unelucidated. Our findings revealed exosomes derived from activated CD4^+^ T cells as important carriers in myofibroblast activation, which is the core element for cardiac fibrotic remodeling. Importantly, we identified that the exosome-enriched miR-142-3p critically mediated fibroblast-to-myofibroblast transition through targeting APC to activate the WNT signaling cascade.

CD4^+^ T cell activation not only plays an important role in cardiac remodeling [8, 9], but also facilitates the development of non-heart diseases, e.g., human immunodeficiency virus infection [32]. Although the monoclonal antibody targeting to CD4^+^ cells has been available [33], its application needs to take caution due to the physiological necessity of CD4^+^ cells in response to inflammatory stress. In this case, it seems necessary to identify the critical downstream mediator of CD4^+^ cell activation-triggered cardiac fibrogenesis. Our present study met the needs to reveal exosome-enriched miR-142-3p as an important mediator for CD4^+^ cell activation-related cardiac fibrosis. Targeting miR-142-3p should be a good alternative for treating cardiac remodeling post-MI.

Exosomes are important carriers for intercellular communication [10]. Besides the miRNAs, other RNAs, DNAs, proteins and small bioactive materials also act as signal mediators in exosome-targeted cells and tissues [11]. In the present study, we cannot preclude the potential contribution of the exosomal materials other than miRNAs. However, consistent with present findings, several lines of evidences have confirmed the negative correlation of miR-142-3p with pathological cardiac remodeling and cardiac dysfunction [34].

Of note, herein we observed that miR-142-3p was downregulated in ventricular tissues post-MI. Under such scenario, several potential explanations might account for how CD4^+^ cell exosome-enriched miR-142-3p exerted pro-fibrotic effects. First, the exosomes were accumulated at peri-infarct region, where exosomal miR-142-3p, without global increase of miR-142-3p in the heart, might suffice to induce fibrogenic behaviors of local fibroblasts. This case may also explain the obvious appearance of fibrosis at the border region of infarcted myocardium. Second, the myofibroblast activation by local exosomal miR-142-3p may release bioactive molecules, such as TGF [35, 36], to trigger hypertrophic response of cardiomyocytes, leading to pathological cardiac remodeling.

Our study indicated that exosomal miR-142-3p directly targeted to and negatively modulated the expression ofAPC, which has been proven to promote β-catenin degradation in the cytoplasm, resulting in the inhibition of the canonical WNT signaling [37]. Additionally, APC protein is also a positive regulator for GSK-β [38], an important protein involved with cardiac remodeling. Thus, the activated CD4^+^ cell-derived exosomal miR-142-3p conferred the pro-fibrotic effects by modulating APC-GSK-β-β-catenin signal cascade.

**Figure 6:**
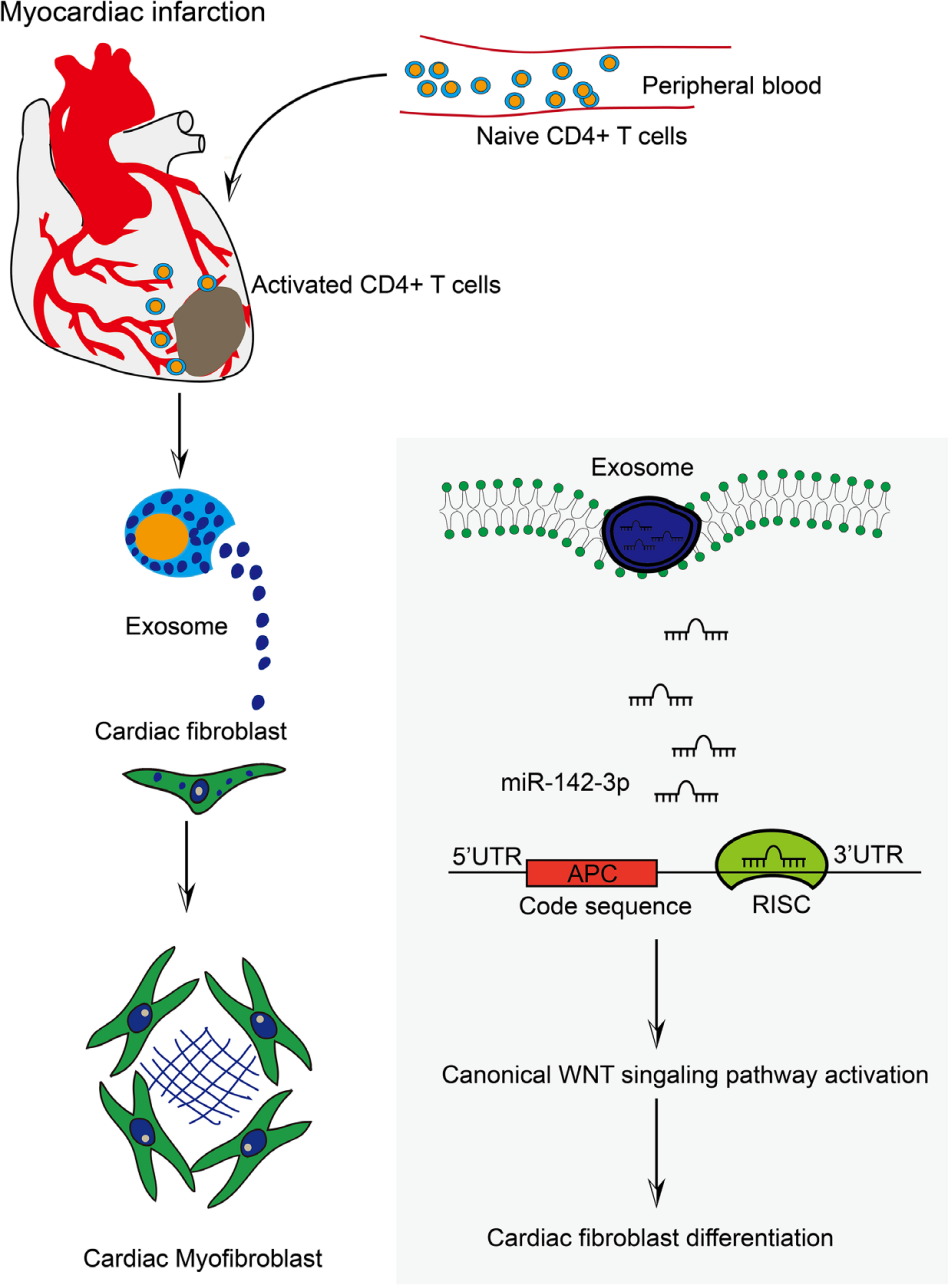
**Activated CD4+ T cells promoted cardiac remodeling via exosomal miR-142-3p-APC-WNT signal axis.** The work model illustrated the action modality of CD4^+^ cells-derived exosomes in myofibroblast activation post-MI. Myocardial infarction induced the accumulation of CD4^+^ T cells into myocardial tissue. Consequently, activated CD4^+^ cells-derived exosomes facilitated CFs transformation through exosomal miR-142-3p-APC-WNT signal axis.

In sum, the exosome derived from CD4^+^ T cells is an important signal carrier in cardiac fibrosis post-MI, and exosomal miR-142-3p serves as the signal conductor (Figure 6). These findings provide new insights into the pathogenesis of MI-related cardiac fibrosis and ischemia-associated heart failure.

## MATERIALS AND METHODS

All experimental procedures on animals were approved by Shanghai General Hospital Institutional Animal Care and Use Committee.

### Animal experiments

A total of 20 healthy male mice (six weeks old, ~ 24 g) were kept under standard housing conditions (temperature, 21 ± 1°C; humidity, 55-60), and provided food and water ad libitum. Either permanent ligation of left anterior descending coronary artery or sham operation was performed as described previously [39]. Mice were randomly assigned into 4 groups including control group (n = 5), sham group (n = 5, mice without ligation of the left anterior descending branch), MI-NC group (n = 5, mice underwent MI and injected with exosomes derived from naive CD4^+^ T cells on the first day post-MI, 40 μg/day by tail vein), and MI-AC group (n = 5, mice suffered MI and injected with exosomes derived from activated CD4^+^ T cells on the first day post-MI, 40 μg/day by tail vein). After injection with Dio-labeled exosomes for 4 weeks, mice were euthanized and the distribution of exosomes in major organs was assessed with IVIS Lumina III In Vivo imaging system (Perkin Elmer, USA).

### Transthoracic echocardiography

Cardiac dimension and function parameters were measured by M-mode echocardiography (Hewlett Packard Sonos 5500, USA). The parameters including left ventricular shortening fraction (LVFS), left ventricular ejection fraction (LVEF), left ventricular end-systolic diameter (LVESD), and left ventricular end-diastolic dimension (LVEDD) were calculated based on the sampled images according to the standard formula [40].

### Histology

Mice were euthanized at the fourth week after MI, and subsequently the hearts were extracted for Masson's trichrome staining. Briefly, hearts were fixed with 4% paraformaldehyde and sliced into 4 μm thickness. Next, the sections at the site of tissue necrosis were stained with Masson's trichrome. The areas of cardiac fibrosis were quantified by Image J software.

### Isolation and culture of primary CD4^+^ T cells and cardiac fibroblasts

Naive CD4^+^ T cells in spleen were purified using a mouse naïve CD4^+^ T cell isolation kit (Invitrogen 8804-6824-74). The purity of the enriched subset was validated by flow cytometry. CD4^+^ T cells were stimulated with plate-bound anti-CD3 (5 μg/ml, BD Pharmingen™, catalog 553057) and anti-CD28 (2 μg/ml, BD Pharmingen™, catalog 553294) for 2 days.

Neonatal mouse cardiac fibroblasts were enzymatically isolated. Briefly, ventricles of neonatal pups (1-2 days old) were minced into 1 mm^3^ pieces, and then were digested with the solution containing 0.1% trypsin and 0.1% collagenase II. The dissociated cells were plated at 37°C for 1.5h, and then the medium was changed to separate the cardiomyocytes from fibroblasts via differential adhesion.

### Flow cytometry

Cells were incubated with APC anti-mouse CD4 (eBioscience, catalog 4329627) on ice for 30 minutes in the dark, and surface staining was performed in FACS buffer (PBS with 2% fetal bovine serum and 2 mM EDTA). All data were acquired on a Fortessa X-20 (BD Biosciences), and live cells were gated for analysis with FlowJo software (Supplementary Figure 1).

### Isolation and characterization of exosomes

Exosomes were isolated by differential centrifugation in conditioned media. Briefly, the conditioned medium was initially cleared of cellular debris, and the dead cells were removed with two sequential centrifugation steps at 2500 g for 10 min at 4°C The cell supernatants were then centrifuged at 110,000 × g for 70 min at 4°C. The pellets were washed with PBS and the ultracentrifugation protocol was repeated. The final exosome pellet was resuspended in PBS. The morphology of exosomes was defined by the transmission electron microscopy (TEM) [41]. The expression levels of exosomal markers TSG101, CD63 and CD81 were assessed by western blotting. Protein amounts in exosomes were quantified using Nanodrop.

### Plasmids and siRNA transfection

Overexpression plasmids with pcDNA3.1-APC were generated by Vigene (Shandong, China). miR-142-3p mimics, miR-142-3p inhibitors and scrambled miRs (miR-NC) were synthesized by Ribobio (Guangzhou, China). For cellular transfection, the isolated cardiac fibroblasts were pre-cultured overnight and then transfected with plasmids or miRNAs using Lipofectamine 3000 (Invitrogen, Cat. No. 11668019) following the manufacturer's recommendations. After 48 h, the cells were subjected to RNA or protein isolation, or immunofluorescence analysis.

### Immunofluorescence assay

After fixation with 4% paraformaldehyde, the cells were permeabilized with 0.5% Triton X-100. Then, the cells were blocked with primary antibody dilution buffer for 1 h at room temperature and incubated with anti-α-SMA primary antibody overnight at 4°C. Primary antibody was removed and the cells were washed twice with PBS, then incubated with Cy3-secondary antibody (1:800, A0516, Beyotime, China) for 2 h at room temperature. The nucleus were stained with DAPI (C1005, Beyotime, China). Fluorescent images were taken on a microscope (Leica Microsystems, Mannheim, Germany).

### Cell proliferation and migration assay

EdU (5-ethynyl-2’-deoxyuridine) staining was used to determine cell proliferation as described previously. As for cells migration assay, transwell chambers with 8-μm pores (Costar) were used following manufacturer’s instructions. Images were taken using a microscope (Leica Microsystems, Mannheim, Germany) at ×200 magnification.

### Dual luciferase activity assay

To identify the target gene of miR-142-3p, the 3' UTR-APC luciferase reporter plasmids containing the wild-type or mutant binding sites of miR-142-3p were constructed. Then, 500 ng of plasmid and 50 nM of miR-142-3p mimic or scrambled control were co-transfected into 293T cells using lipofectamine 3000 (Invitrogen, USA). After transfection for 36 h, the luciferase activity was measured using a Dual Luciferase Report Assay Kit (Promega, USA) according to the manufacturer’s instructions. All assays were done in triplicate and repeated in, at least, two independent experiments.

### Quantitative real-time PCR

Total RNAs were extracted with TRIZOL reagent following the manufacturer's instructions (Takara, Dalian, China). The PrimeScript^TM^ RT reagent Kit (Takara Bio, Shiga, Japan), miRNA reverse transcription kit and miR-142-specific stem-loop primers (Sangon Biotech, shanghai, China) were used to perform the reverse transcription in order to synthesize single-stranded cDNA. qRT-PCR was performed with the SYBR Green PCR Master Mix (TaKaRa, China) using ABI-7300 Real-Time PCR Detection System (Applied Biosystems, USA). U6 and GAPDH were used as internal controls. Primers used for qRT-PCR analysis were listed in Supplementary Table 1.

### Western blot analysis

Total proteins were separated on 7.5% SDS-PAGE at 60-100 V for 2 h, and then transferred to PVDF membrane (Millipore, USA) at 300 mA for 1.5 h. The membranes were blocked with 5% non-fat dry milk for 1.5 h at room temperature. Next, membranes were incubated with primary antibodies at 4°C overnight and secondary antibodies at room temperature for 2 h. The primary antibodies included: rabbit anti-α-SMA (1:1000, ab32575, Abcam), rabbit anti-Col1a1 (1:1000, A16891, ABclonal), rabbit anti-Col3α1 (1:1000, A3795, ABclonal), rabbit anti-β-tubulin (1:1000, #2128, CST), rabbit anti-CD63 (1:1000, ab217345, Abcam), rabbit anti-CD81 (1:1000, ab109201, Abcam), anti-TSG101 (1:1000, ab125011, Abcam), rabbit anti-β-catenin (1:1000, #8480, CST), and rabbit anti-APC (1:1000, ab40778, Abcam). All blots were visualized using the ECL reagent Kit (Millipore Corp, USA). The blot intensities were quantified with Image J software.

### Enzyme-linked immunosorbent assay (ELISA)

The naive CD4^+^ T cells in 96-well plates (1 × 10^4^ cells/well) were treated by anti-CD3/CD28 bodies. After 36 hours, the culture supernatant was collected and concentrations of IL-2, IL-10, IFN-γ and TNF-α were analyzed using ELISA kits (Cusabio, Wuhan, China).

### Statistical analysis

The data were expressed as the mean ± SD and analyzed using SPSS 19.0. Statistical comparisons between 2 groups were performed by a *t*-test. The difference among multiple groups was evaluated using a one-way ANOVA followed by Bonferroni post-tests, and *P* < 0.05 was regarded as statistically significant.

## Supplementary Material

Supplementary Figures

Supplementary Table 1
