# Supplementary material for: Activated CD4+ T cells-derived exosomal miR-142-3p boosts post-ischemic ventricular remodeling by activating myofibroblast
Source: Aging (Albany NY). 2020 Apr 23;12(8):7380–96. doi: 10.18632/aging.103084 (PMC7202529; doi:10.18632/aging.103084)
Supplement: Supplementary Figures [file aging-12-103084-s002..pdf]

SUPPLEMENTARY FIGURES

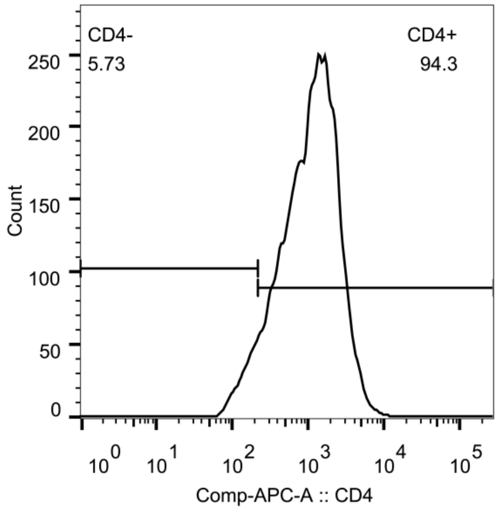

Supplementary Figure 1. The purity of CD4+ T cells. $CD4^+$ T cells in spleen were purified using a mouse naïve $CD4^+$ T cell isolation kit, and the purity of the naive $CD4^+$ T was validated by flow cytometry. The image shown is representative of three independent experiments.

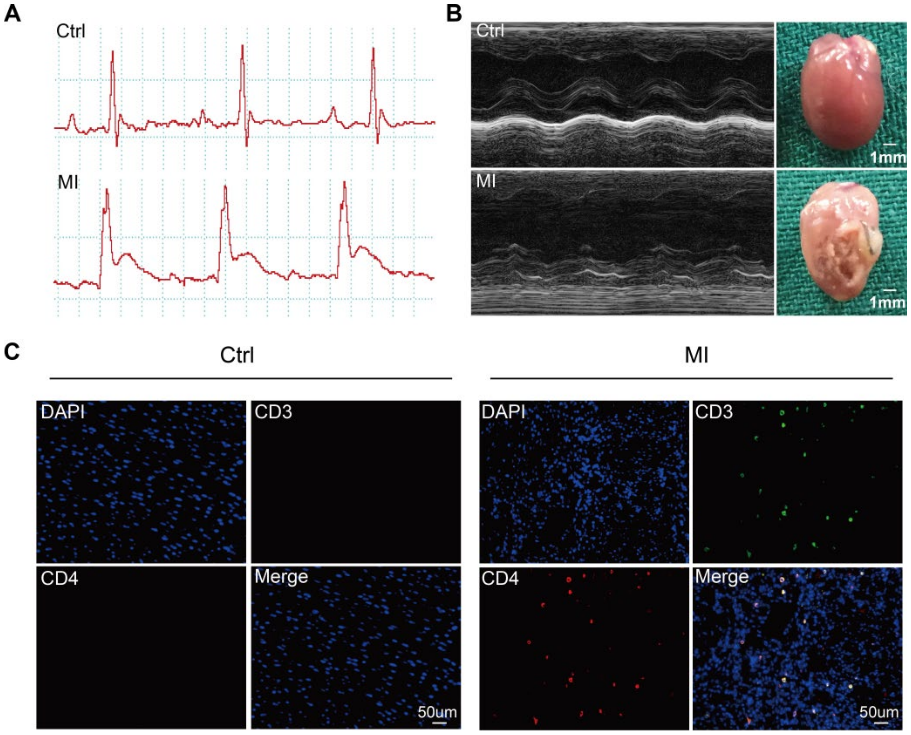

Supplementary Figure 2. $CD4^+$ T cells infiltrated in the heart of myocardial infarction. (**A**) Electrocardiograms showing significant ST-segment elevation by MI. The images shown are representative of three independent experiments. The electrocardiograms shown are representative of three independent experiments. (**B**) Representative echocardiograms and picture of sham-operated and MI mice at four weeks after surgery. n=5 per group. (**C**) Immunofluorescent labeling of $CD4^+$ cells in mice heart at the 4th week post-MI. The images shown are representative of three independent experiments. Ctrl: control. MI: myocardial infarction. Scale bar = 50 μm.

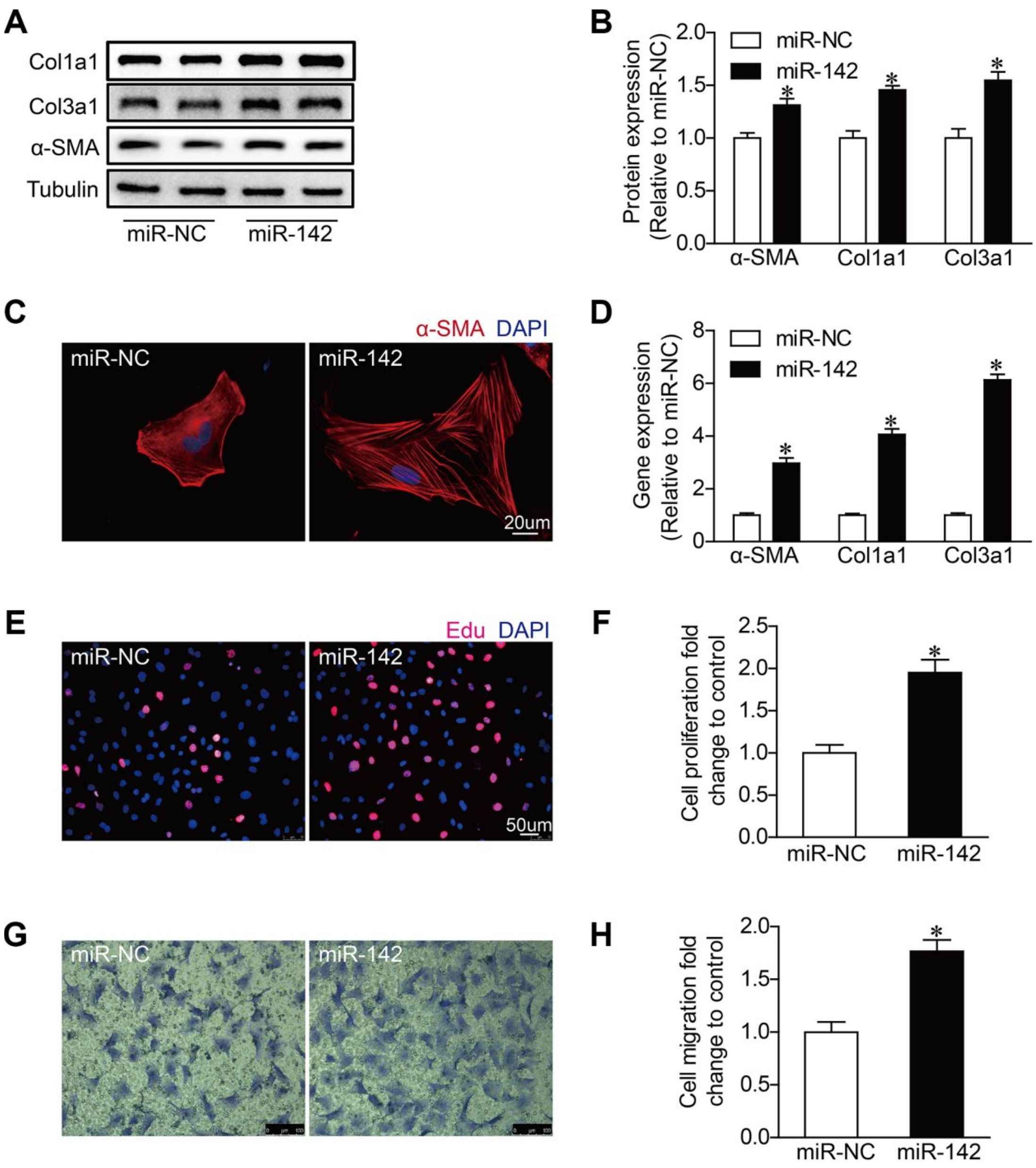

Supplementary Figure 3. MiR-142-3p promoted cardiac fibroblasts differentiation, proliferation and migration. (**A**) Western blotting analysis of α-SMA, Col1a1 and Col3a1 levels in cardiac fibroblasts after transfected with miR-142-3p mimics or miR-NC for 48h. The blots shown are representative of three independent experiments. (**B**) Quantitative analysis of proteins expression. n=3 per group. *P < .05. (**C**) Immunofluorescent analysis of myofibroblast activation. The images shown are representative of three independent experiments. Scale bar = 20 μm. (**D**) qRT-PCR detection of α-SMA, Col1a1 and Col3a1 in cardiac fibroblasts after transfected with miR142-3p mimics for 48h. n=3 per group. *P < .05. (**E**) EdU incorporation detection of cardiac fibroblast proliferation. The images shown are representative of three independent experiments. Scale bar = 50 μm. (**F**) Quantification analysis of cell proliferation using EdU assay data. *P < .05. (**G**) Transwell assay of cardiac fibroblast migration. The images shown are representative of three independent experiments. Scale bar = 100 μm. (**H**) Quantification analysis of cell migration using transwell assay data. *P < .05.
