# Supplementary material for: Activated CD4+ T cells-derived exosomal miR-142-3p boosts post-ischemic ventricular remodeling by activating myofibroblast
Source: Aging (Albany NY). 2020 Apr 23;12(8):7380–96. doi: 10.18632/aging.103084 (PMC7202529; doi:10.18632/aging.103084)
Supplement: Supplementary Table 1 [file aging-12-103084-s001..pdf]

SUPPLEMENTARY TABLE

Supplementary Table 1. Primers used in this study.

Name	Sequence (5'- 3')
α-SMA	Forward: ATCAGGGAGTAATGGTTGGAATG<br>Reverse: GGTCTCAAACATAATCTGGGTCAT
Col1a1	Forward: AGCTTTGTGGACCTCCGGCT<br>Reverse: ACACAGCCGTGCCATTGTGG
Col3a1	Forward: TGAATGGTGGTTTTCAGTTCAG<br>Reverse: GATCCCATCAGCTTCAGAGACT
APC	Forward: GCCTCAGCACTTACCATTCA<br>Reverse: TCCTCTCCTCCGCCACA
β-catenin	Forward: CTTCCAGACACGCCATCATG<br>Reverse: TGGTGATGGCGTAGAACAGT
rno-mir-142-3p RT	UAGCAGCACAUCAUGGUUUACA
mir-142-3p	Forward: CGCGTAGCAGCACATCATGG<br>Reverse: AGTGCAGGGTCCGAGGTATT
miR-142-3p mimics	UAGCAGCACAUCAUGGUUUACA
miR-142-3p inhibitors	UGUAAACCAUGAUGUGCUGCUA
U6	Forward: AGAGAAGATTAGCATGGCCCCTG<br>Reverse: AGTGCAGGGTCCGAGGTATT
β-actin	Forward: TGGCACCACACCTTCTACAAT<br>Reverse: GACCAGAGGCATACAGGGAC
